# A Method to Compensate for the Errors Caused by Temperature in Structured-Light 3D Cameras

**DOI:** 10.3390/s21062073

**Published:** 2021-03-16

**Authors:** Oriol Vila, Imma Boada, David Raba, Esteve Farres

**Affiliations:** 1 Graphics and Imaging Laboratory, University of Girona, 17003 Girona, Spain; 2 Insylo Technologies S.L., 17003 Girona, Spain; david.raba@insylo.com (D.R.); esteve.farres@insylo.com (E.F.)

**Keywords:** RGB-D camera, camera calibration, temperature effect, structured light, infrared pattern distortion

## Abstract

Although low cost red-green-blue-depth (RGB-D) cameras are factory calibrated, to meet the accuracy requirements needed in many industrial applications proper calibration strategies have to be applied. Generally, these strategies do not consider the effect of temperature on the camera measurements. The aim of this paper is to evaluate this effect considering an Orbbec Astra camera. To analyze this camera performance, an experimental study in a thermal chamber has been carried out. From this experiment, it has been seen that produced errors can be modeled as an hyperbolic paraboloid function. To compensate for this error, a two-step method that first computes the error and then corrects it has been proposed. To compute the error two possible strategies are proposed, one based on the infrared distortion map and the other on the depth map. The proposed method has been tested in an experimental scenario with different Orbbec Astra cameras and also in a real environment. In both cases, its good performance has been demonstrated. In addition, the method has been compared with the Kinect v1 achieving similar results. Therefore, the proposed method corrects the error due to temperature, is simple, requires a low computational cost and might be applicable to other similar cameras.

## 1. Introduction

Three-dimensional (3D) shape measurements have become fundamental in many different applications including robotics, virtual reality, industrial inspection or autonomous navigation, just to name a few [1,2,3,4,5]. Different technologies were successfully implemented in the past decades to measure the 3D information of an object, however how to perform these measurements in an efficient, effective and precise manner is still an important focus of research. Among all the technologies that have been proposed, 3D imaging technologies such as stereo vision, structured light and time of flight are the most cost-effective [6]. For a comparison of red-green-blue-depth (RGB-D) cameras representing these three main technologies see [7].

**Figure 1:**
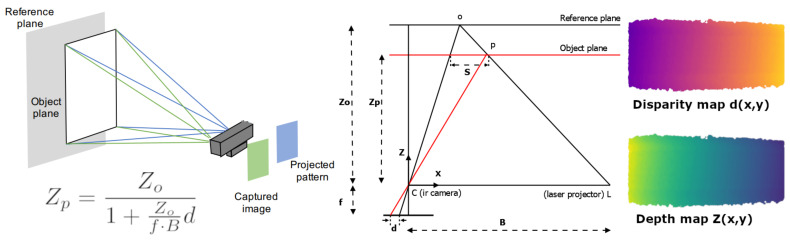
Red-green-blue-depth (RGB-D) camera components and main parameters involved in the disparity and depth map computations. The laser projector L projects a speckle pattern that is registered at a certain distance $Z_{o}$. During a measurement, the sensor compare the position in the image space between the observed points p and its original registered position (disparity). Using triangulation is possible to estimate the depth Zp of such point.

In this paper, our interest is focused on the 3D structured light imaging technology. In this technique, a pattern is projected on a scene and is then captured with a camera from a different position. Since the captured pattern is deformed by the scene shape, the analysis of the disparity from the original projected pattern provides the depth information. As it is illustrated in Figure 1, the basis of this technique is triangulation. Particularly, the depth of a scene point $Z_{p}$ can be computed following the Equation (1) described in [8]

(1) $$Z_{p}=\frac{Z_{o}}{1+\frac{Z_{o}}{f\cdot B}d}$$

that can be rearranged to

(2) $$Z_{p}=\frac{B\cdot f}{d+k}$$

where *B* is the baseline between the camera and the projector, *f* is the focal length from the camera, *d* is the disparity value in the image space between the observed and registered position of *p*, and $k=\frac{f\cdot B}{Z_{o}}$ is a camera constant that depends on the pattern registration distance ($Z_{o}$) and its intrinsic parameters. The disparity value is usually given in pixel-units requiring the conversion of focal length to this unit. Moreover, in the majority of cases, the disparity values are given as horizontal distances as only horizontal displacements of the projector and the camera are performed. The baseline determines the depth range and the depth accuracy. A tutorial on the topic can be found in [9].

In the context of structured light cameras, the Microsoft’s Kinect is the reference one [10,11]. This camera, launched in 2010, returns images like an ordinary camera, but instead of color, each pixel value represents the distance to the point. The camera consists of: (i) an infrared (IR) projector that casts an IR speckle dot pattern into the object; an IR camera that captures the reflected IR speckles, and a color camera. Although, the Kinect was originally conceived for gaming, its low cost, its reliability as well as its good documentation led the camera to be the basis of many innovative applications in the robotic and computer vision community [12]. Moreover, the sensor has been the basis of other RGB-D cameras such as Asus Xtion [13], Orbbec Astra 3D [14] or Occipital Structure Sensor [15]. All them are used in many high accuracy applications, including indoor 3D modeling [16,17,18,19], simultaneous localization and mapping (SLAM) [20,21,22], or augmented reality [23], which require a rigorous calibration and error modeling of RGB-D camera data to produce high quality information [24,25].

Usually, RGB-D cameras are factory-calibrated and the calibration parameters are stored in their internal memory. Unfortunately, this calibration is not accurate enough to satisfy the high-precision requirements of certain applications. Moreover, since deviation and uncertainty of the depth measurement increase with the measurement distance, and the depth measurement error and uncertainty vary across different pixel positions, the depth measurement errors have to be also corrected [26]. To overcome these limitations different strategies to calibrate the intrinsic parameters (focal length, principal point, and lens distortion) of the color and depth cameras and the extrinsic parameters (relative position and orientation) between the cameras have been proposed [8,25,27,28]. For a survey on calibration methods see [29]. Generally, proposed methods are tested on specifically designed scenarios where different parameters such as the distance from a target object, the light conditions or the interference between devices are considered. However, few attention has been given to the errors introduced by the temperature in the obtained results. In this regard, Mankoff and Russo [30], evaluated the Kinect in the context of Earth science research detecting reductions on the accuracy and precision of the measures due to both internal and external thermal changes. Fiedler and Müller [31] described the influence of temperature variations on Kinect’s images and range measurements as well as practical rules to reduce the caused calibration and measurement errors. DiFilippo and Jouaneh [32] compared three different RGB-D cameras examining amongst others the effect of internal temperature on the distance reported by the camera. Microsoft patented a method and an apparatus to compensate for the temperature shifts of a structured light pattern of a depth imaging system [33]. The patent documented the drift of the speckle pattern due to temperature variation of the laser illuminator and also identified the limitations of solutions based on thermoelectric coolers. Its method is based on a temperature compensation model that collects dot pattern images for different ambient temperatures, applies a regression model for each dot position, and recovers a reference view of the pattern for any given ambient temperature before applying stereo searching algorithm for calculating depth information. More recently, Heindl et al. [34] proposed a real-time pixel-wise depth correction method for RGB-D cameras considering both spatial and thermal aspects. The method is based on a Gaussian Process Regression in a four dimensional Cartesian and thermal domain. They also proposed to leverage modern GPUs for dense depth map correction in real-time.

Despite proposed solutions to correct thermal effects on some RGB-D cameras, there are limitations such as the closely guarded secret of proprietary solutions [33] or the required computation cost [34] that hinder the implementation of these solutions to other cameras. Therefore, in case of applications where used sensors do not compensate for the thermal error, specific solutions to mitigate or eliminate it have to be applied. The aim of this paper is to evaluate the effect of temperature on RGB-D camera measurements, focusing on the Orbbec Astra camera manufactured by Orbbec. This camera is used in many industrial applications being a key component of automation, robotics or health care areas, among others [35]. To evaluate the camera performance, an experimental study is presented from which it can be concluded that temperature variation has an undesired effect on the camera’s performance leading to errors in the measured distances. This error can be represented as an hyperbolic paraboloid function. To model it, two different approaches are described, the first one is based on the IR distortion map and the second one on the depth map. After modeling the produced error, a method to compensate for it is proposed. To demonstrate the good performance of the method, this is evaluated in experimental and real conditions and it is also compared with the results obtained with the Kinect v1, considered the reference camera in the context of structured light cameras. Therefore, the main contributions of the paper can be summarized as follows:

1. Two strategies to model the error due to temperature of the depth maps obtained from RGB-D structured light cameras.
2. A method to compensate for the error due to temperature of RGB-D structured light cameras.
3. The evaluation of the proposed method in experimental and real scenarios.

Although the proposed approach has been focused on the Orbbec Astra camera, the obtained results and the approach might be applicable to other similar cameras.

Besides this introduction, the paper has been structured as follows. In Section 2, the preliminary study that has been carried out to evaluate the Orbbec Astra camera when exposed to different temperatures is presented. Then, the two approaches to model the error caused by thermal variations are described as well as the proposed method to compensate for it. The different experiments that have been carried out to test the proposed compensation method are presented and discussed in Section 3. Finally, conclusions and future work are given in Section 4.

## 2. Materials and Methods

### 2.1. Preliminary Study

The study presented here aims to evaluate the RGB-D Orbbec Astra camera performance in front of temperature changes. First, the study set-up will be described and then, a first evaluation of collected data will be given.

#### 2.1.1. Study Set-Up

The study was conducted in a professional thermal chamber with a working volume of 2200×3800×2500 mm. The chamber supported control of temperature within a range of $-50{\;}^{{}^{\circ}}$C to $85{\;}^{{}^{\circ}}$C ± $0.5{\;}^{{}^{\circ}}$C with a temperature rate until $1.5{\;}^{{}^{\circ}}$C/min and a relative humidity of 10% to 90%±2%. The chamber was equipped with a passive air dehumidifier to prevent the condensation of water vapor. Inside the chamber an RGB-D Orbbec Astra sensor was placed on a table in a secure and stable position. The scene of interest was the planar wall of the chamber in front of the sensor. This was covered by a white paper to avoid the metallic effect of the chamber wall. A set of marks placed on the chamber floor were considered to align the camera and the table with the target and ensure perpendicularity. The distance between the camera and the target measured with a laser rangefinder was 1600 mm. No light effects were considered since the light was uniform in all the study. The evaluated temperatures ranged from $60{\;}^{{}^{\circ}}$C to $-20{\;}^{{}^{\circ}}$C decreasing one by one. The Si7021 sensor was used to ensure that the chamber and the sensor had the same temperature. For each sensor capture, the camera identifier, the chamber temperature, the IR image, and the depth map were stored, generating the data for our study.

**Figure 2:**
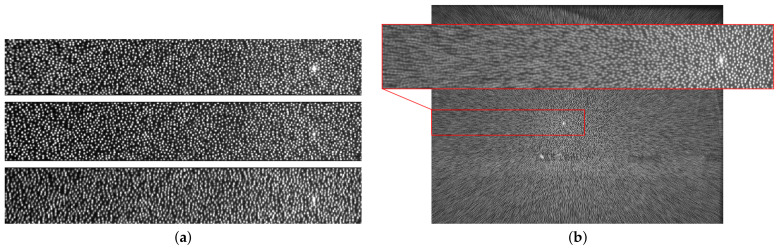
(**a**) From top to bottom, part of the IR images obtained at $-2{\;}^{{}^{\circ}}$C, $28{\;}^{{}^{\circ}}$C, and $58{\;}^{{}^{\circ}}$C. The brightest point in the image indicates the center of the pattern. (**b**) Image corresponding to the mean of the IR images obtained at all evaluated temperatures.

**Figure 3:**
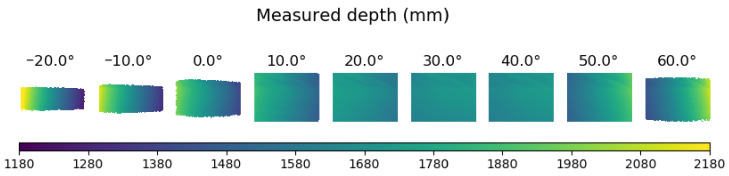
The depth maps obtained at different temperatures. The target object was at 1600 mm.

In Figure 2a, part of the IR images for three different temperatures are shown. It can be seen that temperature variations created distortions on the IR pattern. To better illustrate these distortions, the average image from the sequence of IR images is shown in Figure 2b. It can be observed that the distortion increased with the distance from the center of the IR pattern which corresponded to the brightest point of the image. This effect is due to the distortions on the *x* direction. There were also variations on the *y* direction but these did not affect the *z* computations and for this reason they were not considered. In Figure 3, the depth maps for three different temperatures are shown. The scale color indicates the captured measure. It can be observed that while the real *z* was 1600 mm, the values of the depth maps obtained from the sensor ranged from 1180 mm to 2180 mm. Therefore, temperature *t* had an effect on both the IR image and the depth map.

#### 2.1.2. Modelling the Distortion via IR Images

**Figure 4:**
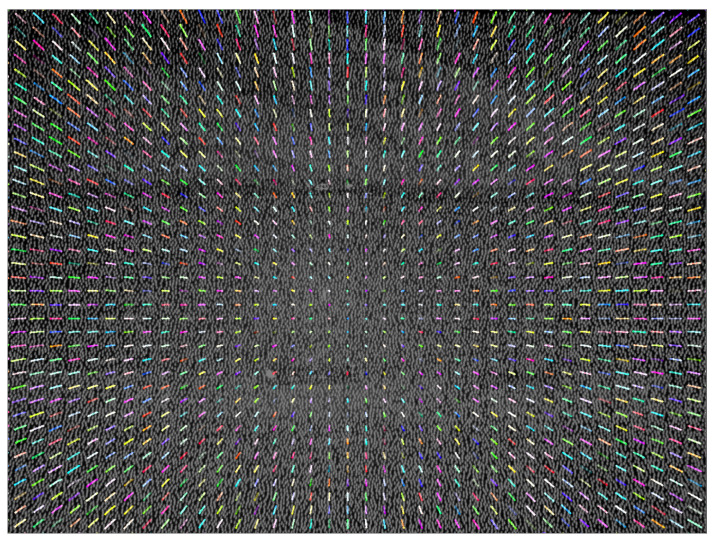
Expansion of the captured IR pattern with the temperature using the optical flow algorithm.

Once the error caused by thermal conditions was detected, our next goal was modeling the distortion of the IR image. To quantify the distortion in the sequence of images, the optical flow algorithm [36] was applied. This algorithm estimated the pixel displacements between a sequence of images using block matching and assuming constant movement in a local neighbourhood of pixels. For this experiment, the motion of 1600 points uniformly distributed across the IR image was tracked. The obtained result is illustrated in Figure 4 where the distortion trace is plotted for every tracked point during the full sweep of temperature.

**Figure 5:**
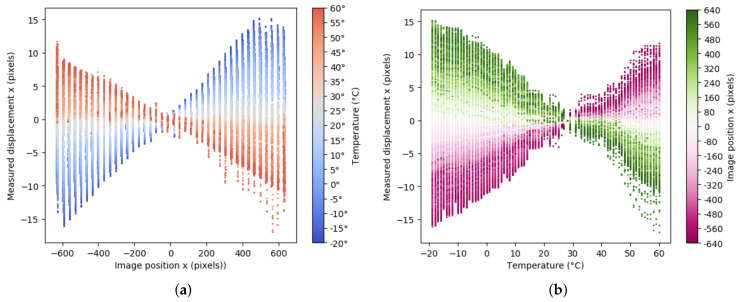
The infrared (IR) image distortion in function of (**a**) the horizontal position in the image and (**b**) the temperature. The different temperatures and displacements are shown using a color scale.

**Figure 6:**
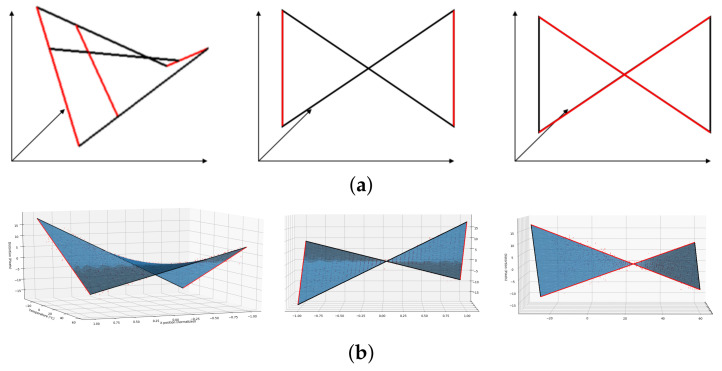
Different views of (**a**) an hyperbolic paraboloid function defined as the union of the lines joining two points moving on two non-coplanar lines and (**b**) the $D_{E}$ function that models the distortion error of the IR images due to temperature changes.

For our purposes, the optimal temperature was defined as the one with the lowest root-mean-square error (RMSE) value, i.e., the one with no distortion on the IR image. Then, the center of this image was taken as the reference point to measure the displacements of the other IR images in the *x* direction for the different temperatures. Taking into account all these considerations, the relationships between: (i) the horizontal distortion displacement and the *x* position and (ii) the horizontal distortion displacement and the temperature, were evaluated and graphically represented in Figure 5a,b, respectively. In this case, the optimal temperature was $28{\;}^{{}^{\circ}}$C. To model the represented error, observe Figure 6a where a hyperbolic paraboloid function is illustrated. It can be seen that this function could be defined as the union of the lines joining two points moving on two non-coplanar lines which can be expressed in terms of two independent linear functions in its slope-intercept form. Comparing the graphical representations obtained from our study (see Figure 5 with Figure 6a), it can be noted that the distortion, $D_{E}$, corresponded to an hyperbolic paraboloid function that depended on the position, *x*, and the temperature, *t*, and can be expressed as

(3) $$D_{E}\left(x,t\right)=\left(w_{1}\cdot x+w_{0}\right)\left(v_{1}\cdot t+v_{0}\right)$$

In this function, variable *x* is the position of the image normalized in the [−1,1] range, variable *t* is the temperature in Celsius degrees, and $w_{1},w_{0},v_{1},$ and $v_{0}$ are the parameters that shape the hyperbolic paraboloid function as two independent linear functions in its slope–intercept form. Moreover, if the *x* coefficient was forced to 1 as in the following expression

(4) $$D_{E}\left(x,t\right)=\left(x+\frac{w_{0}}{w_{1}}\right)\left(w_{1}\cdot v_{1}\cdot t+w_{1}\cdot v_{0}\right)=\left(x+a\right)\left(b\cdot t+c\right)$$

the distortion can be interpreted as a temperature dependent value weighted by the spatial position. The parameter *a* is the intercept at which the spatial position has no distortion and it marks the spatial symmetry. The parameter *b* is the temperature slope in pixels/degree ${}^{{}^{\circ}}$C that establishes the relationship between distortion and the temperature. The parameter *c* in pixels units is the intercept point with the temperature axis and sets the optimal point, i.e., the one with no distortion. Note that $\frac{c}{b}$ is the optimal temperature.

To determine the values of *a*, *b* and *c* parameters, the least squares optimization was applied. The obtained values were: a=0.0406, b=−0.2926 and c=8.6295 modeling the distortion due to the temperature changes as

(5) $$D_{E}\left(x,t\right)=\left(x\right)\left(-0.29t+8.63\right)$$

This function has been represented in Figure 6b.

#### 2.1.3. Relationship between Depth Error and the Disparity Error Obtained from the IR Images

After analyzing the IR image distortion and modeling the error in the disparity domain, which is the physical source of the error, one can already see that the depth map error was going to be dependent of the disparity distortion since the depth map was directly obtained from the disparity map. Particularly, the depth map obtained from the sensor could be described as $Z\left(x,y\right)=\frac{B\cdot f}{d(x,y)+k}$ (see Equation (2)). Since Z(x,y) computation was affected by the disparity error $d_{e}\left(x,y\right)$, the measure obtained by the sensor was not the real Z(x,y) but a $Z_{e}\left(x,y\right)$ such that

(6) $$Z_{e}\left(x,y\right)=\frac{B\cdot f}{d\left(x,y\right)+d_{e}\left(x,y\right)+k}=\frac{B\cdot f}{\frac{B\cdot f}{Z(x,y)}+d_{e}\left(x,y\right)}.$$

It can be observed that the depth error did not only depend on the temperature and the horizontal position, *x*, but also on the measured depth. It can also be observed that for distances close to 0, i.e., Z≈0, the computed $Z_{e}\left(x,y\right)$ was the real one since

(7) $$Z_{e}\left(x,y\right)\approx Z\left(x,y\right)\frac{B\cdot f}{B\cdot f}\approx Z\left(x,y\right).$$

On the contrary, for high distances, i.e., Z>>0, the computed $Z_{e}\left(x,y\right)$ had a great distortion since

(8) $$Z_{e}\left(x,y\right)\approx \frac{B\cdot f}{d_{e}\left(x,y\right)}.$$

**Figure 7:**
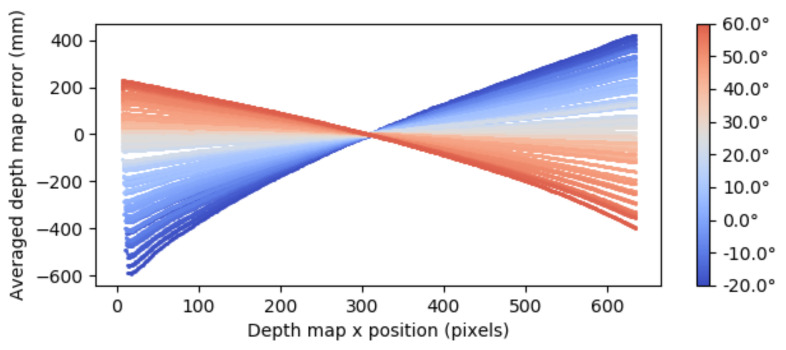
Measured depth error alongside X for different temperatures.

Such a behavior is illustrated in Figure 7 where for a fixed *Z*, the averaged depth map error with respect to the depth map position has been represented. Note that different to the disparity domain that modeled the distortion error in terms of two linearly independent variables (see Figure 5), the error in the depth domain modeled the depth map error in terms of three variables and looses the linearity. Therefore, in order to define a method to reduce the detected error, it made sense to work with the disparity error as it was not only easier to model but also the real source of the problem. Note that although the aim was the correction of the error in the depth map, the correction and the modeling of the error it was carried out on the disparity map, translating depth measures to disparity or vice versa via the $d\left(x,y\right)=\frac{B\cdot f}{Z(x,y)}-k$ relationship.

### 2.2. The Thermal Error Compensation Method

In this section, the method that has been designed to compensate for the error caused by temperature in the RGB-D sensors is presented. This compensation method applies two steps, the first one designed to obtain the distortion model of the sensor, and the second one, to correct the sensor measurements. All the details of both steps are given below.

#### 2.2.1. Step 1. Obtain the Distortion Model of the Sensor

As it has been seen, the thermal distortion can be modeled as an hyperbolic paraboloid function of the form $D_{E}\left(x,t\right)=\left(x+a\right)\left(b\cdot t+c\right)$. Therefore, the first step of the method was designed to obtain the *a*, *b*, and *c* parameters of the function that defined the distortion model. The $D_{E}\left(x,t\right)$ parameters could be obtained by using the least squares optimization approach over a sequence of captures obtained by the the sensor and its associated temperatures. For each capture, the disparity error $d_{e}\left(x,y\right)$ was computed and once all $d_{e}\left(x,y\right)$ have been obtained, the $D_{E}$ could be calculated by applying the least squares optimization approach as

(9) $$D_{E}\left(x,t\right)=\min_{a,b,c}\sum_{n=1}^{N}{\left(d_{e_{n}}\left(x,y\right)-\left(x+a\right)\left(t_{n}\cdot b+c\right)\right)}^{2}$$

where $min_{a,b,c}$ represents the *a*, *b*, and *c* parameters that minimize the function value.

The $d_{e}\left(x,y\right)$ can be obtained from the acquired IR images or from the returned depth maps. The details of both approaches are given in the next.

- *Disparity error computation from IR images.* This approach was based on the method described in Section 2.1.2 which required access to the sequence of IR images and an algorithm to compute the IR image distortion such as the optical flow algorithm. A limitation of this strategy is that it is only applicable when the user has access to the IR images which is not always possible. Moreover, the algorithm used to compute the IR image distortion can also introduce some noise and error in the final results. In addition, it is sensible to lighting conditions requiring fixed contrast, uniform lighting and texture, among others, to properly perform.
- *Disparity error computation from depth map.* The disparity map, d(x,y), could be obtained as $d\left(x,y\right)=\frac{b\cdot f}{Z(x,y)}-k$ (see Equation (2)). Given a depth map Z(x,y) and a reference depth map $Z_{r}\left(x,y\right)$, the disparity error $d_{e}\left(x,y\right)$ can be obtained as the difference between disparities of the reference depth map and the captured depth map, i.e.,
  $$d_{e}\left(x,y\right)=\left(\frac{B\cdot f}{Z_{r}\left(x,y\right)}-k\right)-\left(\frac{B\cdot f}{Z(x,y)}-k\right)$$
  that can be reduced to
  (10) $$d_{e}\left(x,y\right)=B\cdot f\left(\frac{1}{Z_{r}\left(x,y\right)}-\frac{1}{Z(x,y)}\right)$$
  A limitation of this strategy is that it requires an external depth measurement to serve as a reference depth map, $Z_{r}\left(x,y\right)$. However, if this information is not available, any of the captured depth maps can be used as a reference map, in order to obtain a biased $D_{E}$ which can be corrected latter by recomputing the *c* parameter as $c=-t_{opt}\cdot b$. The $t_{opt}$ is a value that can be experimentally obtained by considering different sensors of the same model. For instance, in the case of Orbbec Astra sensors $t_{opt}$ was experimentally set to $30{\;}^{{}^{\circ}}$C. In this way, by assuming a biased reference depth map, the spatial intercept point *a*, the temperature slope *b* and a biased temperature intercept point *c* could be obtained. Then, the *c* parameter was recalculated using the $t_{opt}$ and the correct $D_{E}$ model was obtained. For more details see Section 3.1.
  This second approach was applied to the data obtained in our preliminary study. The obtained $D_{E}$ parameters were a=0.0506, b=−0.2904 and c=8.5035. If we compared these parameters with the ones obtained with the IR images-based approach (a=0.0406, b=−0.2926 and c=8.6295), it can be seen that they were very close. Although both strategies are suitable to model the disparity error, in our experiments this second approach will be used.

#### 2.2.2. Step 2. Correct the Sensor Measurements

The second step of the method was designed to correct the error caused by the temperature in the measurements of the sensor. This error was modeled in the first step of the method as an hyperbolic parabolic function $D_{E}\left(x,t\right)$. From this function, the distortion map for a given *x* and *t* could be obtained. Then, the corrected disparity map, $d_{c}$, for a given *x* and *t* could be obtained as

$$d_{c}\left(x,y\right)=d\left(x,y\right)-D_{E}\left(x,t\right)$$

where the specific distortion map obtained from $D_{E}$ was subtracted from the sensor measured disparity map d(x,y). Note that the measured disparity map could be obtained from the measured depth map using the intrinsic parameters of the sensor as $d\left(x,y\right)=\frac{B\cdot f}{Z(x,y)}-k$. In the same way, the corrected depth map could be generated from the corrected disparity map once the method was applied as $Z_{c}\left(x,y\right)=\frac{B\cdot f}{d_{c}\left(x,y\right)+k}$. Therefore, a depthmap could be corrected using the following equation

(11) $$Z_{c}\left(x,y\right)=\frac{B\cdot f}{\frac{B\cdot f}{Z}-D_{E}\left(x,t\right)}$$

#### 2.2.3. Final Remarks

It is important to notice that the *k* constant, a camera dependent value that may not be known by the user, did not intervene in any of our modeling and correction method. Additionally, in the description of the method, it was seen that the intrinsic parameters were used to perform the computations. However, the method can also be applied when these parameters are not know since it is possible to obtain the information directly from the depth map. Particularly, the expression $Z\left(x,y\right)=\frac{b\cdot f}{d(x,y)+k}$ can be used both to obtain the distortion model of the sensor and to correct the sensor measurements. The disparity error of the measurements can be computed from Equation (10) and the distortion model from

$$D_{E}\left(x,t\right)=B\cdot f\cdot D_{E}^{\prime }\left(x,t\right)$$

where $D_{E}^{\prime }\left(x,t\right)$ is the distortion model without the intrinsic parameters and B·f is just a multiplying factor that gives physical meaning to the model parameter values. If the proposed method is applied to a depth measurement using $D_{E}^{\prime }$, it can be seen that

$$d_{c}\left(x,y\right)=\frac{B\cdot f}{Z(x,y)}-B\cdot f\cdot D_{E}^{\prime }\left(x,t\right)$$

and

$$Z_{c}\left(x,y\right)=\frac{B\cdot f}{B\cdot f\left(\frac{1}{Z(x,y)}-D_{E}^{\prime }\left(x,t\right)\right)}$$

that can be reduced to

$$Z_{c}\left(x,y\right)=\frac{1}{\frac{1}{Z(x,y)}-D_{E}^{\prime }\left(x,t\right)}$$

where the intrinsic parameters are no longer needed. Therefore, the proposed method did not depend on the intrinsic parameters of the sensor.

In the description of the method, it was also considered that the acquired images were from a static scene and hence a single reference image, $Z_{r}\left(x,t\right)$, was required. If the method has to be applied in a dynamic scene, a different reference map for each one of the captures will be required. Therefore, the proposed method supports both static and dynamic scenes.

## 3. Results and Discussion

In this section, the four experiments that were carried out to test the proposed compensation method are presented.

### 3.1. Evaluation Considering Different Cameras

The first experiment was designed to evaluate the robustness of the method when considering other RGB-D Orbbec Astra sensors. Since, in some applications the sensors provided by the manufacturer needed to be modified to fit the scenario conditions, sensors with different configurations will be considered. In particular, in the experiment five different RGB-D Orbbec Astra sensors were evaluated, two of them maintained the original manufacturer configuration, the other two were modified by removing the original plastic case that protects them from the exterior, and in the last one, the original case was replaced by a custom one and also the original peltier was disconnected to eliminate the manufacturer temperature stabilizer. The experiment was conducted in the same professional thermal chamber used in the preliminary study and with the same conditions (see Section 2.1.1). The sensors were placed on a table in a secure and stable position and its temperature was monitored using the temperature sensor (Si7021). The evaluated temperatures ranged from $-20{\;}^{{}^{\circ}}$C to $60{\;}^{{}^{\circ}}$C increasing one by one. For each camera capture, the camera identifier, the chamber temperature, the IR image, and the depth map were stored.

To model the distortion caused by temperature, the values of *a*, *b*, and *c* parameters for each sensor were determined using the least squares optimization approach. The obtained values are presented in Table 1. From these results, it can be observed that parameter *a* was very close to 0, and parameter *b* and *c* ranged between −0.27 and −0.32 and 7.59 and 10, respectively. Therefore, the optimal temperature for these sensors could be set to $t_{opt}=30.0{\;}^{{}^{\circ}}$C. Note that the parameters obtained for the sensors that have not been modified (camera #1 and camera #2) are different. This is due to the fact that although sensors are factory calibrated it is almost impossible that they perform exactly in the same way. For our purposes, the error due to this initial calibration was accepted since its magnitude was minimal compared to the errors due to temperature.

**Figure 8:**
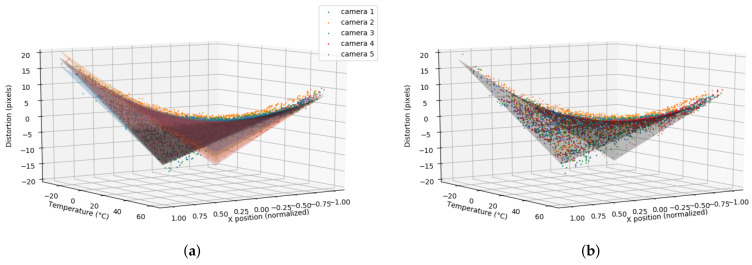
(**a**) The temperature model regression for the five evaluated Orbbec Astra cameras. Each camera has been represented in a different colour. The colour dots correspond to the distortion measurements whereas the surface represents the hyperbolic paraboloid function that has been obtained using the least squares approach. (**b**) The distortion model obtained from the average of all specific camera models.

In Figure 8a, the $D_{E}$ function that models the distortion caused by temperature for each one of the evaluated sensors has been graphically represented. Note that although the specific equation for a camera could be computed from these values, it made sense to consider a default model that can be directly applied to all Orbbec Astra sensors. This could be obtained, for instance, by considering the average values of the obtained parameters for all the cameras. In this way,

(12) $$D_{E}\left(x,t\right)=\left(x\right)\left(-0.3t+9.1\right)$$

can be considered as a global model to be applied to this camera model (see Figure 8b). The cost of using the global model instead of the specific one should be minimal as the parameters were quite consistent between multiple cameras. Therefore, it made sense to consider a default distortion model if a previous evaluation with a reduced number of sensors from the same manufacturer was considered.

### 3.2. Evaluation in Real Environments

The second experiment was designed to evaluate the proposed compensation method in a real scenario and considering two different cameras. To carry out the experiment, a planar target was set on the ceiling room and the Orbbec Astra sensor was placed on the floor at 2100 mm perpendicular to the target. The distance was measured using a laser rangefinder. The room temperature was changed using an electric heater and opening the windows. The sensor captures were done every 10 min during approximately 24 h. For each capture, the time value, the room’s temperature, and the depth map were registered. The obtained data was analyzed measuring the RMSE before and after applying the correction model. To model the error three approaches were considered: (i) using the specific camera parameters, (ii) using the global parameters obtained from the same camera models (see Equation (12)), and (iii) using the camera parameters calculated from the obtained data.

**Figure 9:**
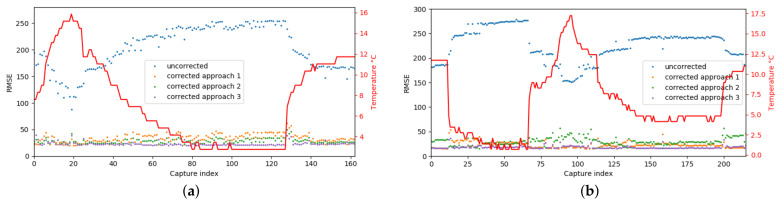
The RMSE before and after applying the correction model to the samples obtained from the testing scenario for (**a**) experiment 1 and (**b**) experiment 2. The red plot corresponds to the temperature values, the blue one to the original depth values and the orange, green and violet plots to the corrected depth values using the three approaches to model the error based on camera parameters (approach 1), global model equation (approach 2) and recomputed camera parameters (approach 3), respectively.

The obtained results for the first camera, after and before applying the correction model, and considering the three error model computation approaches are illustrated in Figure 9a. The red plot corresponds to the temperature values, the blue one to the original depth values and the orange, green and violet plots to the corrected depth values using the three approaches, respectively. Focusing on original depth values (blue plot), it can be observed that, as in the experimental study, the error increased when the temperature moved away from the optimal one. While the real depth was 2100 mm the obtained values from the sensor ranged from 1660 mm to 2740 mm achieving a maximum RMSE of 255.1 mm. On the contrary, focusing on the values obtained after applying the compensation method, it can be observed that, for all the approaches, the RMSE was considerably reduced and the correlation with the temperature was lost. Particularly, the maximum RMSE was 62 mm and it was achieved with the specific parameters approach. Note also that the results obtained with the global parameters approach were slightly better than the ones obtained with the specific camera parameters approach. A reason for this behaviour could be a possible bias of the specific camera parameters that was compensated for when considering the models of all the cameras in a single one.

In this experiment, the temperature variation was 13 degrees and the parameters of the error model were a=0.059, b=−0.36 and c=9.91. These values were similar to the ones obtained with the global approach (see Equation (12)). From this observation, it was considered that it was possible to generate a correction model with just 13 degree variation and a consistent number of samples of at least one point per degree. However, it had to be taken into account that the more the temperature variation and the considered samples, the more robust the correction model was. This is due to the fact that with more measurements the noise in the temperature measurements decreased and a better generalization of the temperature range was obtained.

The same experiment was repeated with a second camera on a different day. The obtained results are illustrated in Figure 9b. Although, the obtained results were similar to the ones obtained with the first camera, in this case, the specific camera parameters approach performed slightly better than the global based approach. Therefore, comparing both approaches, no conclusions on which was the best one could be given. However, it was expected that the global model performed better when the measurements used for the distortion modeling were noisy or biased since it averaged the measurements between multiples cameras leading to more accurate results. For both cameras, it can be seen that the best results were obtained when the distortion was modelled using the camera parameters calculated from the captured data. Note that in this case the distortion and the correction models were computed in the same temperature conditions which was the ideal situation.

### 3.3. Comparison with the Kinect’s Method

The third experiment was designed to compare the results of the Orbbec Astra sensor using the proposed method with the results of the Kinect sensor. This last was considered the reference sensor in the context of spectral imaging RGB-D sensors. It has integrated a method to reduce errors due to temperature [33]. To carry out the experiment, the environment of the previous experiment was considered. A planar target was set in a room and both cameras, the Orbbec Astra and the Kinect, were placed at 2100 mm from the target. The distance was measured using a laser rangefinder. Three captures at 5.52${}^{{}^{\circ}}$, 11.72${}^{{}^{\circ}}$ and 19.30${}^{{}^{\circ}}$ were performed to evaluate the consistency of the method across different temperatures. For each device, the RMSE was measured and in the case of Orbecc sensor, this was computed before and after applying the proposed thermal compensation method. The obtained results are presented in Table 2. It can be observed that, as was expected, the error before applying the compensation method of Orbbec Astra sensor was higher than the error of the Kinect device. However, if the error was compared after applying the proposed compensation model a similar accuracy to the Kinect’s one was achieved. Therefore, the results of the proposed compensation method were comparable to the Kinect’s one.

### 3.4. Comparison with Non-Structured Light Cameras

The last experiment was designed to compare the proposed approach with cameras from other technologies. In particular, the Intel L515 [37] that used LIDAR and Intel D415 [38] that used active IR stereo were considered. These cameras were not subject to the temperature pattern distortion observed in the Orbbec Astra camera. Therefore, it was expected that our approach obtained the same results.

**Figure 10:**
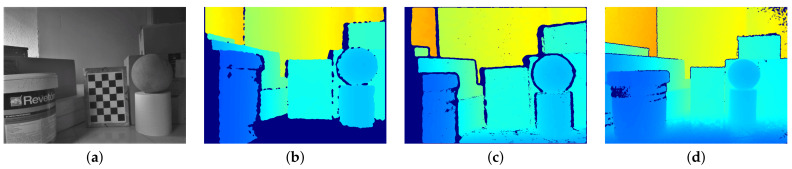
(**a**) The testing scenario and the depth maps obtained with (**b**) Orbecc Astra, (**c**) D415, and (**d**) L515 cameras.

**Figure 11:**
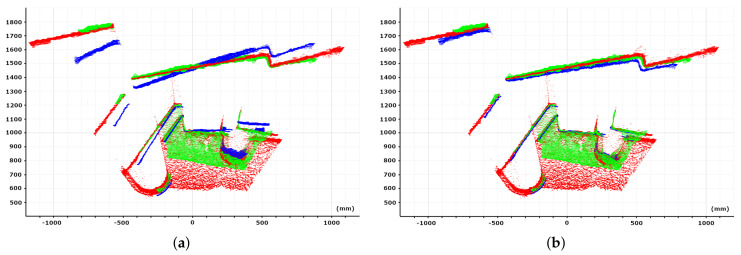
Blue, green and red colors correspond to the 3D world points acquired with the Orbbec Astra, the Intel D415, and the Intel L515 when (**a**) no correction and (**b**) correction is applied to the Orbbec Astra camera.

To carry out the experiment, the scene illustrated in Figure 10 with objects of different shapes and dimensions placed on the floor were captured from the same position using the three cameras. The measured ambient temperature was $11{\;}^{{}^{\circ}}$C. For comparison purposes, a chessboard pattern was placed in the scene and was used to register the 3D position of the different cameras. The obtained results are represented in Figure 11a where blue, green, and red colors correspond to the 3D world points acquired with the Orbbec Astra, the Intel D415, and the Intel L515, respectively. The same information after applying the proposed approach to the Orbbec Astra is represented in Image (b). It can be seen that our method was able to adjust the scene geometry, being consistent with the results of Intel cameras. Moreover, taking the L515 camera result as the reference one, the closest point-to-point correspondence RMSE of the Orbbec Astra was computed. The obtained values before and after applying the correction method were 47.6 and 14.5 mm, respectively. Note that in this case, if the distortion was not corrected, a measurement error up to 180 mm, corresponding to the farthest surface of the scene, could be reached. As was expected, with our correction method, the obtained results were comparable to the state-of-the-art cameras.

### 3.5. Limitations

Although the different experiments that have been carried out show the good performance of the proposed approach, there are some limitations that have to be taken into account.

The first one is no comparison of our method with state of the art methods other than the one proposed for the Kinect sensor. Generally, proposed methods consider variable target distances to test their proposals while in our case a fixed depth has been considered in all the experiments since no information of this variable is required to model the error. This has been a limiting factor, since using these data to reproduce the experiments leads to overfitting results that cannot be considered as representative. To overcome this limitation, as a future work, a new set of experiments with different depth values will be considered. However, if the comparison is done in terms of number of measures required to create the correction model, our approach is simpler since it only requires a single static scene while the other methods need measures taken at different distances. Moreover, our distortion model only requires of three parameters which can be obtained using a simple least squares optimization. Because of this simplicity the correction computation cost is minimal ($4.35\times 10^{-3}$ s per depth map using CPU) while the other methods [34] require up to 20 s per depth map and also the use of GPUs to accelerate computations in order to obtain real time results. In addition, if the comparison is done in terms of adaptability, the proposed approach can be easily adapted to other cameras.

The second limitation is related to the no calibration of intrinsic camera parameters. Since the error due to temperature is one order of magnitude higher than the one due to intrinsic parameters (for instance, a temperature error can reach more than 200 mm RMSE while the error due to intrinsic parameters is about 10 mm RMSE on distance measurements of 2000 mm), it has been considered that it is not necessary to consider the effect of this calibration in our experiments. However, for future experiments, it will be interesting to take into account this information.

The last limitation is related to the temperature measurements when modeling or correcting the distortion. The useful temperature corresponds to the projector element of the camera and it can differ from the ambient temperature because of two reasons: (i) the warm-up of the device when it is powered on [39] (internal electronic heat dissipation) and (ii) the heat isolation and slow thermal inertia of the camera (because of its plastic case). It has been tried to minimize these effects by reducing the time between power-on and the capture and increasing the time between a temperature variation and a capture. However, there is no guarantee that the temperature of the camera and the one detected by the temperature sensor are exactly the same which can result in bias and errors. Therefore, to ensure a good accuracy, all these possible sources of errors need to be considered and reduced when possible.

## 4. Conclusions and Future Work

In this paper, a two-step method to compensate for the error in the measurements of low cost structured light cameras caused by temperature is presented. The first step models the error which, after evaluating the sensor performance in a thermal chamber, is modeled as an hyperbolic paraboloid function. To obtain the parameters of this function two strategies have been proposed, one based on the captured infrared images, and the other on the depth maps. The second step of the method corrects the error of the measurements. The well-performance of the proposal is demonstrated in the different experiments that have carried out using the Orbbec Astra sensor. The proposed method is simple and can be applicable to other similar cameras. Future work includes the design of new experiments in real world applications considering more features that can influence the method performance and other sensors.

## Figures and Tables

**Table 1:** The values of the parameters obtained with the least squares optimization.

Camera	*a*	*b*	*c*	Optimal Temperature
1	0.03	−0.27	7.60	28.13
2	0.06	−0.31	10.32	32.75
3	0.04	−0.30	8.90	30.05
4	−0.02	−0.31	9.94	31.97
5	0.05	−0.29	8.70	30.40

**Table 2:** RMSE values obtained for the Kinect and the Orbbec Astra sensor in mm before and after applying the proposed thermal compensation method and considering captures at three different temperatures.

Camera	RMSE (5.52°)	RMSE (11.72°)	RMSE (19.30°)
Kinect	33.59	28.8	23.51
Astra	217.75	158.1	103.96
Astra corrected	32.72	22.50	27.42

## Data Availability

Data available on request.
